# Estrogen Metabolism Pathways in Pregnancy and Subsequent Breast Cancer Risk: A Prospective Follow-up Study

**DOI:** 10.21203/rs.3.rs-8022341/v1

**Published:** 2025-11-17

**Authors:** Rebecca Troisi, Roni Falk, Helja-Marja Surcel, Saila Kauppila, Tuomas Mirtti, Mika Gissler, Joshua Sampson, Xu Xia, Shaoqi Fan, Gretchen Gierach

**Affiliations:** National Cancer Institute, National Institutes of Health; National Cancer Institute, National Institutes of Health; University of Oulu; Oulu University Hospital and Oulu University; Helsinki University Hospital; THL Finnish Institute for Health and Welfare; National Cancer Institute, National Institutes of Health; Cancer Research Technology Program, Leidos Biomedical Research, Inc.; National Cancer Institute, National Institutes of Health; National Cancer Institute, National Institutes of Health

**Keywords:** pregnancy, maternal hormones, estrogen metabolism, estrogen and progesterone receptor negative breast cancer

## Abstract

**Background:**

In the years following pregnancy, breast cancer risk is elevated, particularly for hormone receptor negative (HR-) tumors. Exposure to high maternal circulating estrogens, when the breast is vastly remodeling in structure and morphology, has been associated with risk, particularly for HR-tumors. Estrogen metabolite profiles in nonpregnant women, notably the ratio of 2:16 hydroxylation (OH) pathway metabolites, are associated with postmenopausal breast cancer development; whether estrogen metabolism during pregnancy influences subsequent HR- breast cancer risk is unknown.

**Methods:**

Weconducted a population-based case-control study in women 19–39 years identified in the Finnish Maternity Cohort Biobank and linked with the Finnish Cancer Registry to identify breast cancer diagnoses within 20 years of pregnancy. Estrogens and metabolites were measured using highly reliable and sensitive LC-MS/MS methods in serum collected during the first and second trimesters of pregnancy. Included were invasive, ER-/PR- breast cancer cases (n=449) and controls (n=449) matched on maternal age at index pregnancy, parity, calendar year of serum collection, gestational week of blood collection, and number of freeze/thaw cycles. Associations between estrogens and breast cancer risk were estimated using odds ratios (ORs) with 95% confidence intervals (CIs) from conditional logistic regression models.

**Results:**

The median years of follow-up between blood collection and breast cancer diagnosis/control selection was 9 (range 0–19). Total estrogens were positively associated with ER-/PR- breast cancer (OR associated with a doubling of total estrogens 1.16; 95% CI 1.02–1.32), as were metabolites in the 16-pathway including estriol [OR 1.11; 95% CI 1.01–1.22], 16-epiestriol [OR 1.11; 95% CI 1.01–1.21)], 17-epiestriol [OR 1.06; 95% CI 1.01–1.13], and total 16-hydroxylation pathway metabolites [OR 1.11; 95% CI 1.00–1.24]. There was no clear association with the ratio of 2:16 hydroxylation pathway metabolites. Some associations differed by parity, age at diagnosis, and gestational timing of blood collection, but interactions were not statistically significant. Results were similar when restricted to cases occurring within 15 years since pregnancy.

**Conclusion:**

This prospective study demonstrated positive associations of estrogen metabolites in pregnancy and risk of ER-/PR- breast cancer, but the magnitudes varied by metabolite. No strong or consistent pattern for one metabolic pathway emerged suggesting that total estrogen concentrations during pregnancy are associated with subsequent HR- breast cancer development, regardless of how they are metabolized.

## Introduction

Breast cancer risk shows a transient increase in the years following pregnancy.^1^ While most tumors are estrogen receptor (ER) positive (+), the risk of ER negative (−) tumors with poorer prognosis^2,3^ remains elevated for decades after delivery as compared to nulliparous women.^1,3^

The increased breast cancer risk immediately following pregnancy may be mediated by exposures experienced during pregnancy, when the breast vastly remodels in structure and morphology preparing for lactation. Maternal pregnancy estradiol (E2) concentrations have been associated with risk in young women who subsequently developed breast cancer.^4–7^ The direction of the associations with E2 appears to depend on age at breast cancer diagnosis, with an elevated risk at younger ages and a reduced risk at older ages^8^ and may be limited to hormone receptor negative (HR-) breast cancer cases.^5^

Estrogen is primarily metabolized in the liver through hydroxylation then methylation and glucuronidation. Competitive estrogen metabolic pathways result in different total and relative quantities of pathway metabolites. The effect of estrogen concentrations on breast cancer risk may depend on metabolic pathway. In postmenopausal women, elevated 16-hydroxylation pathway metabolites have been associated with increased breast cancer risk and metabolites from the 2-hydroxylation pathway have been associated with reduced risk,^9^ although studies have not evaluated the association with tumor receptor status, and the interpretation of findings for urinary estrogen metabolites and risk in premenopausal women is less clear.^10,11^ While estrogen metabolism in pregnancy occurs in the placenta and fetal adrenal glands and liver, in addition to the maternal adrenal glands, many of the metabolites are similar to those in the non-pregnant state and all enter the maternal circulation (**additional** Fig. 1).

We hypothesized that maternal estrogen metabolism profiles assessed in pregnancy are positively associated with risk of HR- breast cancer development. We conducted a prospective, nested case-control study of HR- breast tumors occurring up to 19 years after pregnancy in the Finnish Maternity Cohort (FMC). This long-term study represents a rare opportunity to characterize biomarkers in pregnancy in relation to risk of an aggressive tumor subtype in younger women.

## Methods

### Study design

The FMC was established in 1983 and is a nationwide serum bank with approximately 2 million serum samples collected during the first and early second trimester of pregnancy (5th to 95th percentile: months 2–4 of pregnancy) from over 950,000 women. The FMC covers virtually all pregnancies in Finland during 1983–2016 with archived prenatal serum specimens drawn for routine screening for congenital infections (HIV, Hepatitis B and syphilis). After registration of each new pregnancy presenting before the second trimester, a blood sample was drawn after informed consent for routine screening tests; remaining serum specimens (usually 1–3 mL for each pregnancy) were stored at −25°C in a protected, central repository the Biobank Borealis of Northern Finland for research purposes. Using a national Personal Identification Number (PIN) given to all Finnish Citizens and permanent residents, data enabling follow-up were retrieved through linkages with nationwide registries, including the Finnish Population Registry (for emigration and vital status), Finnish Medical Birth Registry (MBR; 1988–2015), and Finnish Cancer Registry (1991–2015). Maternal and pregnancy characteristics were available from the FMC and supplemented by the MBR. Maternal body mass index (BMI) was based on the mother’s prepregnancy weight and height (weight/height^2^), and the smoking variable represents any smoking during the pregnancy.

### Eligible sample

Breast cancer cases and controls were selected from women with pregnancies at age 18–39 years registered in the FMC with blood sampled at < 20 weeks’ gestation (exact day of blood sampling is recorded and available). Eligible for study were women who had singleton pregnancies, had data on year of blood collection and gestational week at blood collection, and whether samples had been previously thawed.

### Hormone receptor-negative Breast cancer cases

To identify breast cancer cases, the FMC was linked with the Finnish Cancer Registry, where reporting has been mandated by legislation since 1961 and which covers nearly 100% of diagnosed cancers in Finland.^12^ Cases were restricted to women with histologically-confirmed invasive breast cancer (ICD-10: C50) with no history of *in situ* breast cancer or other invasive cancer (except nonmelanoma skin cancer) before their breast cancer diagnosis. Included in the analysis were cases diagnosed with ER-/PR- tumors within 19 years after their most recent pregnancy that occurred before cancer diagnosis (referred to as the index pregnancy, i.e., the pregnancy that provided the blood sample). Neither the medical birth registry nor the FMC had information on HR status. Therefore, we searched for potentially eligible cases at five university hospitals (where cases are typically treated) and randomly selected cases who had a sample in the FMC and met the required inclusion criteria.

Controls were chosen from eligible women in the FMC with no prior diagnosis of *in situ* breast cancer or invasive cancer (except nonmelanoma skin cancer) before their matched case’s date of cancer diagnosis (reference date). Controls from the eligible cohort were matched to each case on maternal age at index pregnancy (± 1 year), parity (primiparous [first pregnancy], number of births), blood sample collection calendar year (± 1 year), and gestational week (within 1 week) of blood collection. We additionally matched on number of sample freeze/thaw cycles. Controls were required to be alive and living in Finland at their matched case’s diagnosis date (to ensure they would be registered in the cancer registry had they been diagnosed with cancer). Each control was selected randomly from the eligible pool.

In 2015, there were 15,295 breast cancer cases with at least one serum sample in the FMC. Of these, we included in the analysis were 449 ER-/PR- cases and 449 matched controls meeting the inclusion criteria for this study.

### Laboratory assays

The FMC was assembled for the standardized collection and analysis of pregnancy biospecimens. Seventy-four percent of cases and controls were perfectly matched on freeze/thaw cycles, 63.9% and 81.1%, respectively had samples with no previous thawing, and 36.1% and 18.9% were previously thawed.

The stable isotope dilution high-performance liquid chromatography tandem mass spectrometry (HPLC-MS/MS) was used to quantify total (conjugated, including both glucuronidated and sulfated, plus unconjugated) concentrations of 15 estrogens (the two parent estrogens, estrone (E1) and estradiol (E2), and 13 estrogen metabolites.^13,14^ The case and control samples were assayed in matched sets, in random order within batches. Blinded quality control samples from a high estrogen concentration and a low estrogen concentration pool of blood from pregnant women were inserted within each batch (10%). Coefficients of variation for samples from both pools were < 2% and intraclass correlation coefficients were > 99% for all the metabolites (**additional** Table 1).

### Statistical analysis

We compared maternal and perinatal characteristics in cases and controls and tested for differences using t-tests and Wilcoxon rank-sum tests for continuous covariates and Chi-squared tests for categorical variables. All estrogens were natural log-transformed to normalize their distributions and were also classified into quartiles based on their distributions among controls. Sum of estrogens by pathway, ratio of pathway to total estrogens, and ratio of 2:16 hydroxylation pathways were calculated.^9^ We also applied log_2_ transformation to all continuous estrogens to determine the doubling effects of an individual estrogen metabolite concentration on breast cancer risk.^5^ Conditional logistic regression estimated odds ratios (OR) and 95% confidence intervals (CI) for breast cancer risk (dependent variable) associated with variables representing the estrogen exposures (i.e., total estrogen concentrations [parent estrogens and metabolites], parent estrogens [E1 and E2], estriol [E3], individual metabolites, and the ratio of pathway/total estrogens, including 2:16 hydroxylation pathways). Regression models were repeated with further adjustment for E2 (E2 was significantly and positively correlated with the other estrogens (**additional** Fig. 2)). We reviewed the associations between breast cancer risk and quartiles of estrogens to assess whether associations appeared monotonic, and then we reviewed the associations between breast cancer risk with estrogen measures of doubling concentration.

In subgroup analyses, we stratified the associations of the estrogens and breast cancer risk by parity (primiparous, i.e., the woman was in her first pregnancy resulting in a live birth; multiparous), age at diagnosis (< 40; ≥40 years), and gestational week at blood draw (median ≤ 10;>10). In addition, analyses were performed restricting to cancer cases and matched controls whose diagnosis/reference date was within 15 years after index pregnancy and for cancer diagnosis < 50 years of age. Whether associations of the individual estrogens and breast cancer risk differed by these factors was assessed by including an interaction term (e.g., E2*parity) in the model; p < 0.05 was considered statistically significant.

*A priori* hypotheses included associations of breast cancer risk with total and parent estrogens, and the ratio of 2:16 hydroxylation pathways. While statistical significance was defined as two-sided P < 0.05, or a CI not including 1.0, for associations that were not *a priori* and subgroup analyses, we relied less on statistical significance for interpretation and instead described patterns of results. .

## Results

### Case and control characteristics

The distributions for the matching variables, maternal age at index pregnancy, parity, and blood sample collection calendar year, and gestational week of serum specimen collection are shown in Table 1. In addition, age at first pregnancy, maternal smoking, and newborn sex and birth weight did not differ between cases and controls. Most of the participants were missing information on BMI (84%) since the MBR data collection was started in 2004. Among cases (n = 57; 13%) and controls (n = 87; 19%) with existing data, there was no statistical difference in BMI (P-Wilcoxon rank-sum test = 0.33). Age at breast cancer diagnosis was relatively young (median 40.6 years) because of the year the MBR was established (1987), ranging from 25.7–55 years. The median number of years between the index pregnancy and cancer diagnosis was 9 and ranged from > 0 to 19 years.

### Relative percentages of estrogen metabolites and comparison between ER-/PR- breast cancer cases and controls

Table 2 shows the distributions of the estrogen metabolites (pg/mL) comparing cases with controls. Median concentrations of the total and parent estrogens, E1 and E2, appeared higher in cases than controls, but the differences did not reach statistical significance. Likewise, median pathway metabolites generally were higher in the cases than controls, for example, total 16-pathway metabolites (median 1153.1 and 1031.4; p = 0.11), and E3 (median 789.5 and 693.3; p = 0.11) in the 16-pathway. 4ME2 (4-Methoxyestradiol, median 1.6 and 1.5; p = 0.029 from the 4-pathway and 16-Epi3 (16-Epiestriol, median 71.7 and 61.5; p = 0.048) from the 16-pathway were the only two individual estrogen metabolites that were statistically significantly higher in cases than controls. The individual pathways as a percentage of total estrogens, as well as the ratio of the 2:16 pathways did not statistically significantly differ between cases and controls.

#### Odds ratios for ER-/PR- breast cancer associated with estrogens

**Figure 2:**
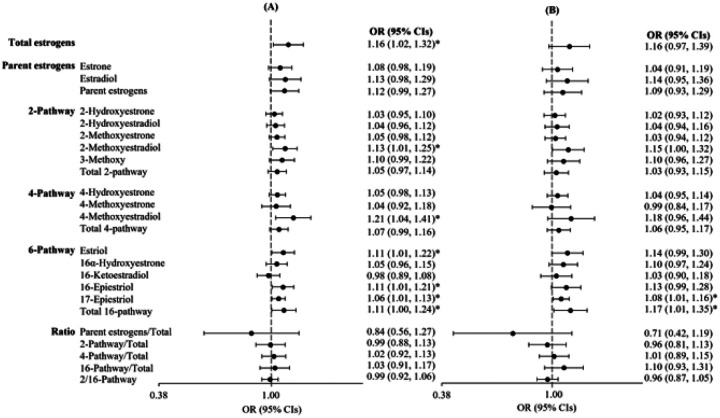
Forest plots of odds ratios (ORs) and 95% confidence intervals (CIs) for ER-/PR- breast cancer associated with maternal pregnancy serum individual estrogen metabolites and metabolism pathways (A) before and (B) after adjustment for estradiol (continuous and natural log-transformed), Finnish Maternity Cohort (n=449 cases and n=449 controls). ORs (black circle) are shown on a log2 scale and black solid lines represent 95% CI. Associations are from conditional logistic regression models. *Denotes associations between individual estrogen metabolites and breast cancer risk at alpha=0.05 significance level.

Figure 2 presents the OR for a doubling of pregnancy estrogen concentration and ER-/PR- breast cancer risk overall and separately adjusted for total E2. Many of the associations between the parent estrogens, estrogen metabolism pathways, and estrogen metabolites were positive for breast cancer risk including total estrogens (OR 1.16; 95% CI 1.02–1.32), 2ME2 (2-Methoxyestradiol OR 1.13; 95% CI 1.01–1.25), 4ME2 (4-Methoxyestradiol OR 1.21; 95% CI 1.04–1.41), E3 (Estriol OR 1.11; 95% CI 1.01–1.22), 16EPIE3 (16-Epiestriol OR 1.11; 95% CI 1.01–1.21), 17EPIE3 (17-Epiestriol OR 1.06; 95% CI 1.01–1.13), and total 16-hydroxylation pathway metabolites (OR 1.11; 95% CI 1.00–1.24). Notably, breast cancer risk was not associated with the ratio of 2:16 hydroxylation pathways (OR 0.99; 95% CI 0.92–1.06). None of the ORs changed more than 7% with adjustment for E2. In the model for E3, we additionally adjusted for E1 as well as E2 to determine if an inverse association resulted as demonstrated in another study.^7^ The OR for E3 and breast cancer risk adjusted for E2 was 1.08 (95% 0.97–1.21) and 1.09 (95% CI 0.98–1.21) when adjusted for E2 and E1 (data not shown).

The overall results were similar when the analysis was restricted to < 15 years between maternal pregnancy sample collection and HR- breast cancer diagnosis (e.g., OR associated with total estrogens was 1.19, 95% CI 1.03–1.37 even after excluding 64 cases and matched controls; **additional Fig. 3**), and when further restricted to < 10 years (excluding an additional 104 cases and matched controls (data not shown).

### ORs for estrogens and ER-/PR- breast cancer by selected participant characteristics

**Additional Fig. 4–6** presents the OR for a doubling of pregnancy estrogen concentration and breast cancer risk in subgroup analyses. Most associations between the estrogens and breast cancer remained positive and similar to the overall results although the magnitude varied partly owing to changes in sample size. The association between total 16-hydroxylation pathway metabolites and breast cancer risk was stronger in multiparous women (OR 1.17; 95% CI 1.01–1.35) than primiparous women (1.05; 0.91–1.23) (**additional Fig. 4**). The OR for E2 and breast cancer risk was greater among women diagnosed at < 40 years than at ≥ 40 years (OR 1.22; 95% CI 1.00–1.48 and 1.05; 0.87–1.27, respectively), and similarly for 4ME2 ((4-Methoxyestradiol OR 1.43; 95% CI 1.13–1.82 and 1.06; 0.86–1.30, respectively) (**additional Fig. 5**); results among women < 50 years of age at cancer diagnosis were similar to those overall. Conversely, other estrogens showed stronger associations among women diagnosed at ≥ 40 years of age as compared with breast cancer diagnosed at < 40 years (e.g. for E3 [Estriol] OR 1.16; 95% CI 1.01–1.34 and 1.06; 0.93–1.21, respectively). There appeared to be small differences in the results by whether blood samples were collected at ≤ 10 weeks’ gestation (n = 384; ≤ 10 week) or > 10 weeks’ gestation (n = 514; >10 week), with generally stronger associations in the former for the parent estrogens (e.g., for E2 [Estradiol] OR 1.22; 95% CI 1.01–1.46 and 1.03; 0.84–1.26, respectively) and total metabolites (OR 1.23; 95% CI 1.03–1.47 and 1.08; 0.89–1.31, respectively) (**additional Fig. 6**). In comparing the characteristics of women whose blood was drawn earlier in gestation compared with later, we observed a few small differences in their age at cancer diagnosis (mean 40.2; SD 6.1, and 41.6; 6.4 years, respectively, = 0.016), years from pregnancy blood collection to cancer diagnosis (mean 8.2; SD 4.9, 9.2; 5.4, respectively, p = 0.049); and whether the sample had not been previously thawed (69% and 75.1%, respectively, p = 0.043). Restricting the analyses to nonsmokers did not materially change our observed associations (data not shown).

## Discussion

In this prospective study of maternal serum estrogen metabolites and ER-/PR- breast cancer risk up to two decades after pregnancy, we found largely positive associations that remained after adjustment for total E2 and when analyses were restricted to fewer years since last pregnancy. The 16-hydroxylation pathway metabolites appeared to be more consistently associated with elevated risk although there was no clear evidence indicating that associations were stronger for any one metabolic pathway. There was no association between the ratio of 2:16 hydroxylation pathways and breast cancer risk. Some associations of the estrogens with breast cancer appeared stronger in subgroups defined by parity, age at breast cancer diagnosis, and timing of blood draw in gestation, but none of the interactions were statistically significant. These subgroup analyses lacked power and were viewed as exploratory and requiring confirmation in later studies.

Previous studies have demonstrated associations of maternal parent estrogens including E2 and E1,^4–7^ progesterone,^4,5^ and testosterone^5,6^ with breast cancer risk in the years following pregnancy, but the findings overall and in subgroups are mixed, and they have not examined associations with estrogen metabolites in addition to E3.^4–7^ Two other studies have used FMC data to evaluate estrogens and breast cancer risk.^5,8^ In the first (n = 536 cases and n = 1,049 controls), maternal E2 concentrations from primiparous pregnancies were not associated with overall breast cancer risk but were positively associated with risk of breast cancer diagnosed at < 40 years of age (upper quartile OR, 1.81; CI, 1.08–3.06) while inversely associated with cancer diagnosed at ≥ 40 years (upper quartile OR, 0.64; CI, 0.40–1.04).^8^ Fortner et al.^5^ in a larger study using FMC data (n = 1,199 cases and n = 2,281 controls) confirmed the positive association of E2 in primiparous pregnancies with breast cancer diagnosed in women age < 40 years [OR for 4th vs. 1st quartile 1.60; 95% CI 1.07–2.39), and the inverse association of E2 in women diagnosed at 40 years or later [4th vs. 1st quartile OR 0.71 (CI, 0.51–1.00)]; however, risk in the former was restricted to ER-/PR- cases. Our data suggested that elevated E2 was more strongly associated with increased ER-/PR- risk among women 40 + years at diagnosis compared with < 40 years at diagnosis, although the interaction by age at cancer diagnosis was not statistically significant. In a third study using Northern Sweden Maternity Cohort data (n = 223 cases and n = 417 controls),^6^ there were no associations between maternal E2, free E2, and E1 in primiparous pregnancies with ER-/PR- breast cancer. These studies sampled blood in primiparous pregnancies only^5,6,8^ while we sampled blood in the last pregnancy before cancer diagnosis. Our results for E2 among women who had only one pregnancy (blood was drawn in the first pregnancy which was also the last pregnancy before diagnosis) were similar to results among multiparous women.

This is the first study of which we are aware to investigate the relationship of estrogen metabolite profiles in pregnancy with subsequent breast cancer risk. We restricted our study to ER-/PR- breast cancers which are more likely to occur in this younger age group and closer to the pregnancy, and because of the previous observation that circulating E2 in young women was only associated with risk of ER-/PR- breast tumors.^5^ Moreover, less is known about the etiology of these often aggressive cases.^15^ While we observed a general positive pattern of association between the estrogens and breast cancer risk, our results did not support our hypothesis that the ratio of 2:16 hydroxylation pathway metabolites would be inversely associated with breast cancer risk, suggesting that elevated maternal serum estrogens, regardless of metabolic pathway, increase risk of ER-/PR- breast cancer.

There was a suggestion that the associations of 16-pathway metabolites in early pregnancy and ER-/PR-breast cancer risk were more consistent than for the other pathways. Two other studies analyzed third trimester concentrations of the 16-pathway metabolite E3. One study^4^ found no association and the other^7^ found an inverse association for E3 but only when adjusted for E2 and E1; we did not observe an inverse association in our study. Besides different timing of the blood collection in pregnancy, we used HPLC-MS/MS while other studies used radioimmunoassay and our CV for E3 was lower than in the other two studies (0.41% vs. 9%^4^ and 4.7%^7^). In contrast to the other major estrogens in pregnancy, E2 and E1, approximately 90% of E3 precursors originate from the fetus and are metabolized in the placenta.^16^ If the positive association of ER-/PR- breast cancer risk with 16-pathway metabolites, including E3, that we observed were confirmed, it could indicate that the circulating concentrations responsible do not depend on the mother’s capacity to metabolize estrogens.

Although not statistically significant, positive associations for some of the estrogens were stronger among women with blood collected ≤ 10 weeks of pregnancy than at 10 weeks or later. Women who discovered they were pregnant and presented for blood draw earlier in gestation may have differed in breast cancer risk factors. There were some small differences between women who had their blood drawn earlier compared with later in gestation regarding age at cancer diagnosis, years between most recent pregnancy and cancer diagnosis, and whether the sample had been previously thawed. However, there was little evidence of effect modification by these factors in our results.

Parity is an established and strong risk factor for ER + tumors with a reduced risk among parous women compared with nulliparous women long after pregnancy, and at older ages when breast cancer usually occurs. The association with parity is weaker for ER- tumors.^15^ In contrast, breast cancer risk is elevated soon after pregnancy,^1^ when women are younger, and ER- tumors comprise a greater percentage of total breast cancers than among older women.^17^ Estrogens and other steroid and growth hormones rise precipitously in pregnancy and are critical in preparing the mammary gland for lactation.^18^ Dramatic changes to mammary tissue begin in the first weeks of pregnancy with major epithelial cell proliferation. Developing alveoli replicate to form lobuloaveolar units which undergo terminal differentiation later in pregnancy. Reduced susceptibility to carcinogenesis of molecularly reprogrammed mammary cells is hypothesized to explain the long-term beneficial effect of pregnancy.^19^ Conversely, highly elevated growth hormones, primarily estrogens, could explain the initiation of carcinogenic changes in epithelial tissue, or progression of already established cancer.^20^ We were interested in exploring whether maternal estrogen metabolite concentrations played a role in this elevated risk. Our results demonstrating mainly positive associations between estrogens and ER-/PR- breast cancer risk lasting almost 20 years after pregnancy suggest that pregnancy’s influence on carcinogenesis is more important than previously thought.

Estrogen concentrations in early pregnancy may be correlated with levels after the pregnancy and throughout life. Within-woman positive estrogen correlations would be consistent with maternal exposure as a proxy for exposure more proximate to cancer development. To evaluate this hypothesis would be difficult, requiring a cohort of women with repeated blood collections in pregnancy and throughout life.

Neither the Finnish Maternity Cohort nor the Finnish Medical Registry has information on breastfeeding. Breastfeeding has been associated with a reduction (about 12%−25%) in risk for premenopausal breast cancer most notably for ER- disease (Palmer et al., 2019).^21^ However, in Finland, 99% of mothers breastfeed their infants.^22^ Most of the participants were missing information on BMI (84%) since the MBR data collection was started in 2004, so we were unable to assess its impact on the risk associations. The degree of missingness in BMI did not significantly differ by case status making it less likely that not adjusting for BMI could have biased our estimates of association between estrogen concentrations and breast cancer risk. Similarly, smoking could be a potential confounder; however, most of the cases (86.5%) and controls (88.4%) did not report smoking during pregnancy and results did not change when analyses were restricted to nonsmokers. We did not have information on stage at diagnosis of the breast cancer tumors. Finland has a long history of breast cancer screening by mammography beginning in the 1970s. Currently, screening is biennial among women 50–69 years. Only 6.9% of cases in our study were over 50 years of age at diagnosis. Together with high participation in the screening program (greater than 80%), it is unlikely that any differences in screening by case status biased the results. Nichols, et al.^1^found that the association of breast cancer risk in the years after pregnancy was greater in women with a family history of breast cancer than among those who did not. We did not have information on family cancer history; therefore, we were unable to directly address whether family history influenced the associations between estrogen metabolism and breast cancer risk.

The coefficients of variation based on quality control samples dispersed within the batches were very low, indicating excellent estrogen metabolite assay reproducibility. Nonetheless, random misclassification in the estrogen measurements could cause bias toward null findings. The effects of specimen handling and storage on hormone measurements (sex-steroids, pregnancy-specific hormones and insulin-like growth factor) in FMC samples have been evaluated.^23^ The variation of all estrogen concentrations studied followed the kinetics reported for early pregnancy, and bench-lag time (the time between sample collection and freezing for storage) did not materially affect concentrations. Time to processing has recently been reviewed for many analytes^24^ and steroid hormones appear stable up to 72 hours in serum. Other studies suggest that steroid hormones are not sensitive to freeze/thaw cycles;^25^ most samples were not previously thawed, and cases and controls were matched on cycles and calendar year of blood draw.

## Conclusion

In conclusion, we found evidence that concentrations of total estrogens in pregnancy were positively related to ER-/PR- breast cancer risk up to two decades following pregnancy. In addition, elevated estrogen byproducts resulting from all three metabolic pathways were associated with increased ER-/PR- breast cancer risk; however, the relative amounts of 2-pathway metabolites to 16-hydroxylation pathway metabolites, which have been previously linked to postmenopausal breast cancer risk, were not. Taken together, these data suggest that pregnancy estrogens are associated with ER-/PR- breast cancer regardless of how they are metabolized.

## Supplementary Material

Supplementary Files

This is a list of supplementary files associated with this preprint. Click to download.

- AdditionalTablesandFigures.docx

## Figures and Tables

**Figure 1:**
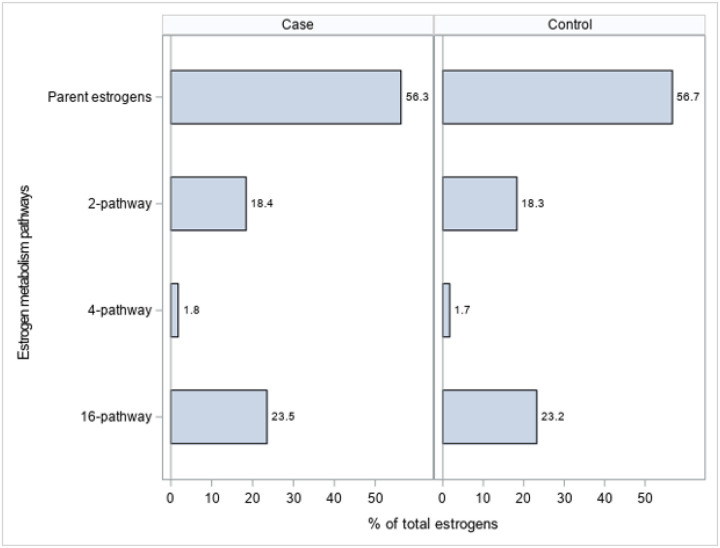
Relative percentages of maternal pregnancy serum concentrations of the parent estrogens (estradiol, estrone) and total 2-, 4-, and 16-hydroxylation pathway estrogen metabolites in controls (N=449) and ER-/PR- breast cancer cases (N=449), Finnish Maternity Cohort.

**Table 1:** Distributions of characteristics of ER-/PR-breast cancer cases and controls matched on previous pregnancies, maternal age at index pregnancy, gestational week at pregnancy, calendar year at index pregnancy, Finnish Maternity Cohort (N = 898)

	Total (n = 898)	ER-/PR- Cases (N = 449)	Controls (N = 449)	
Characteristics	n (%)	n (%)	n (%)	p†
**Previous pregnancies (< index pregnancy)**				0.99
Primiparous*	374 (41.7)	187 (41.7)	187 (41.7)	
1	321 (35.8)	161 (35.9)	160 (35.6)	
2	162 (18.0)	81 (18.0)	81 (18.0)	
3	35 (3.9)	17 (3.8)	18 (4.0)	
≥ 4	6 (0.7)	3 (0.7)	3 (0.7)	
**Age at first birth (year)**				0.93
<25	174 (20.8)	90 (21.6)	84 (20.0)	
25–29	324 (38.7)	161 (38.6)	163 (38.8)	
30–34	260 (31.1)	126 (30.2)	134 (31.9)	
35–45	79 (9.4)	40 (9.6)	39 (9.3)	
Mean (Std)	28.8 (4.6)	28.7 (4.6)	28.9 (4.6)	0.56**
Median (Range)	28.8 (16.9, 44.6)	29.0 (16.9, 40.6)	28.8 (16.9, 44.6)	0.55‡
Missing	61	32	29	
**Maternal age at index pregnancy**				1.00
<25	62 (6.9)	31 (6.9)	31 (6.9)	
25–29	248 (27.6)	124 (27.6)	124 (27.6)	
30–34	354 (39.4)	177 (39.4)	177 (39.4)	
35–41	234 (26.1)	117 (26.1)	117 (26.1)	
Mean (Std)	31.7 (4.3)	31.7 (4.3)	31.7 (4.3)	0.94**
Median (Range)	28.8 (16.9, 44.6)	31.8 (18.7, 40.6)	31.6 (18.1, 40.8)	0.95‡
**Gestational week at index pregnancy**				0.91
< 8	71 (7.9)	34 (7.6)	37 (8.2)	
8–12	589 (65.6)	297 (66.2)	292 (65.0)	
12–17.8	238 (26.5)	118 (26.3)	120 (26.7)	
Mean (Std)	10.9 (2.4)	10.9 (2.4)	10.8 (2.4)	0.93
Median (Range)	10.4 (5.7, 17.6)	10.4 (5.9, 17.6)	10.4 (5.7, 17.6)	0.90‡
**Calendar year at index pregnancy**				0.98
1988–1992	249 (27.7)	124 (27.6)	125 (27.8)	
1993–1998	217 (24.2)	108 (24.1)	109 (24.3)	
1999–2002	207 (23.1)	102 (22.7)	105 (23.4)	
2003–2015	225 (25.1)	115 (25.6)	110 (24.5)	
**Age at breast cancer diagnosis**				
< 35		82 (18.3)		
35–39		125 (27.8)		
40–44		113 (25.2)		
45–49		98 (21.8)		
50–57		31 (6.9)		
Mean (Std)		41.0 (6.3)		
Median (Range)		40.6 (25.7, 57.5)		
**Years from index pregnancy to cancer diagnosis**				
< 5		115 (25.6)		
5–10		136 (30.3)		
11–14		127 (28.3)		
15–19		71 (15.8)		
Mean (Std)		8.8 (5.2)		
Median (Range)		9.0 (0.0, 19.0)		
**Calendar year at cancer diagnosis**				
Q1:1991–2004		122 (27.2)		
Q2: 2005–2008		106 (23.6)		
Q3: 2009–2012		123 (27.4)		
Q4: 2013–2015		98 (21.8)		
**BMI at index pregnancy (kg/m** ^**2**^ **)**				0.95§
< 18.5	4 (2.8)	1 (1.8)	3 (3.4)	
18.5–24.9	94 (65.3)	38 (66.7)	56 (64.4)	
25.0–29.9	34 (23.6)	14 (24.6)	20 (23.0)	
≥ 30.0	12 (8.3)	4 (7.0)	8 (9.2)	
Mean (Std)	24.0 (4.5)	24.3 (4.4)	23.8 (4.6)	0.57**
Median (Range)	23.2 (17.3, 40.9)	23.5 (17.6, 40.0)	23.1 (17.3, 40.9)	0.33‡
Missing	754	392	362	
**Smoking status**				0.45
Never	632 (87.5)	275 (86.5)	357 (88.4)	
Ever	90 (12.5)	43 (13.5)	47 (11.6)	
Missing	176	131	45	
**Neonate’s sex**				0.59
Male	391 (51.4)	170 (50.3)	221 (52.3)	
Female	370 (48.6)	168 (49.7)	202 (47.8)	
Missing	137	111	26	
**Neonate’s birth weight (quartile, grams)**				0.46
570–3320	175 (24.2)	71 (22.6)	104 (25.4)	
3325–3600	162 (22.4)	67 (21.3)	95 (23.2)	
3600–3860	187 (25.8)	80 (25.5)	107 (26.1)	
3860–5100	200 (27.6)	96 (30.6)	104 (25.4)	
Mean (Std)	3611 (486.4)	3648 (461.4)	3583 (503.4)	0.08**
Median (Range)	3620 (570, 5220)	3640 (2240, 5220)	3600 (570, 5100)	0.14‡
Missing	174	135	39	

Matching factors were maternal age at index pregnancy (± 1 year), parity (primiparous [first pregnancy], number of births), blood sample collection calendar year (± 1 year), and gestational week (within 1 week) of blood collection.

* Primiparous woman’s pregnancy is her first.

** P-values from t-tests.

† P-values calculated from Chi-Squared tests except where noted. P-values < 0.05 are in bold font.

‡ P-values from Wilcoxon rank-sum test.

§ P-value from Fisher’s exact test.

**Table 2:** Distributions of maternal pregnancy serum estrogen metabolites overall and by ER-/PR- breast cancer case-control status, Finnish Maternity Cohort (N = 898)

Estrogen metabolites (pg/mL)	Total (n = 898)	ER-/PR- Cases (n = 449)	Controls (n = 449)	p†	p‡
	Median (Range)	Median (Range)	Median (Range)		
**Total estrogens**	5600.2 (801.9, 39223)	5911.2 (1037, 37628)	5517.5 (801.9, 39223)	0.11	**0.03**
**Parent estrogens**					
E1	1569.1 (184.2, 11954)	1591.7 (184.2, 11872)	1523.9 (203.8, 11954)	0.22	0.14
E2	1591.2 (270.9, 9505)	1671.1 (270.9, 8730)	1540.3 (296.1, 9505)	0.18	0.09
Total parent estrogens	3199.2 (499.9, 19317)	3347.4 (600.2, 19317)	3058.6 (499.9, 18907)	0.17	0.08
**2-Pathway**					
2-Hydroxyestrone	437.1 (15.5, 3403)	446.7 (15.5, 2977)	424.7 (25.2, 3403)	0.35	0.49
2-Hydroxyestradiol	61.3 (6.4, 812.2)	63.6 (8.3, 812.2)	59.7 (6.4, 546.8)	0.36	0.36
2-Methoxyestrone	380.6 (11.6, 4478)	402.2 (16.6, 4212)	364.6 (11.6, 4478)	0.26	0.17
2-Methoxyestradiol	60.6 (5.7, 788.7)	63.3 (7.8, 788.7)	58.7 (5.7, 688.2)	0.18	**0.03**
3-Methyl ether	5.3 (1.0, 64.9)	5.6 (1.0, 57.55)	5.0 (1.0, 64.89)	0.14	0.09
Total 2-pathway	993.9 (66.3, 7340)	1058.6 (79.9, 7340)	952.8 (66.3, 7338)	0.19	0.22
**4-Pathway**					
4-Hydroxyestrone	75.3 (3.8, 894.6)	77.2 (3.8, 797.0)	71.1 (4.1, 894.6)	0.20	0.15
4-Methoxyestrone	4.5 (1.0, 30.5)	4.5 (1.0, 30.5)	4.4 (1.0, 18.3)	0.61	0.52
4-Methoxyestradiol	1.5 (0.4, 9.5)	1.6 (0.4, 9.5)	1.5 (0.4, 9.2)	**0.03**	**0.02**
Total 4-pathway	81.8 (6.7, 900.8)	84.4 (6.7, 803.0)	77.3 (6.8, 900.8)	0.17	0.10
**16-Pathway**					
E3	737.4 (36.6, 18238)	789.5 (36.6, 12424)	693.3 (52.9, 18238)	0.11	**0.04**
16α-Hydroxyestrone	154.3 (13.4, 3898)	160.2 (13.4, 1597)	147.3 (17.3, 3898)	0.33	0.33
16-Ketoestradiol	63.9 (7.1, 1310)	62.4 (8.1, 520.0)	65.0 (7.1, 1310)	0.91	0.58
16-Epiestriol	66.2 (2.2, 631.6)	71.7 (2.2, 581.3)	61.5 (3.4, 631.6)	**0.05**	**0.03**
17-Epiestriol	13.4 (0.0, 463.1)	14.2 (0.8, 362.6)	12.2 (0.04, 463.1)	0.08	**0.03**
Total 16-pathway	1083.5 (88.2, 22864)	1153.1 (88.2, 14553)	1031.4 (129.9, 22864)	0.11	**0.04**
**Pathway/Total estrogens (%)**					
Parent estrogens	56.9 (22.7, 84.4)	56.6 (27.3, 84.4)	57.3 (22.7, 81.7)	0.52	0.43
2-Pathway	18.1 (2.1, 42.6)	18.4 (2.2, 40.2)	18.0 (2.1, 42.6)	0.86	0.94
4-Pathway	1.4 (0.1, 9.0)	1.5 (0.2, 9.0)	1.4 (0.1, 8.5)	0.70	0.64
16-Pathway	20.2 (3.0, 74.6)	20.6 (4.8, 70.3)	20.1 (23.00, 74.6)	0.69	0.67
**Ratio of 2/16-Pathway**	0.9 (0.03, 7.5)	0.9 (0.03, 6.2)	0.9 (0.03, 7.5)	0.70	0.78

† P-values were from Wilcoxon rank-sum test using natural logarithm transformed estrogen metabolites. P-values < 0.05 are in bold font.

‡ P-values are from random effect models adjusting for continuous gestational week at blood collection.

**Table:** Abbreviations

**Parent estrogens**
E1 = Estrone
E2 = Estradiol
**2-Pathway**
2OHE1 = 2-Hydroxyestrone
2OHE2 = 2-Hydroxyestradiol
2ME1 = 2-Methoxyestrone
2ME2 = 2-Methoxyestradiol
ME3 = 3-Methoxy, also known as 2-methoxyestrone-3-methyl ether
**4-Pathway**
4OHE1 = 4-Hydroxyestrone
4ME1 = 4-Methoxyestrone
4ME2 = 4-methoxyestradiol
**16-Pathway**
E3 = Estriol
16αOHE1 =16α-Hydroxyestrone
16KETO =16-Ketoestradiol
16EPI =16-Epiestriol
17EPI =17-Epiestriol
**Total Pathway**
Parent
2-Pathway
4-Pathway
16-Pathway
**Ratio of Pathway(s)**
Parent/Total
2-Pathway/Total
4-Pathway/Total
6-Pathway/Total
2/16-Pathway

## Data Availability

The data used in this analysis is deposited in the protected biorepository at the Biobank Borealis of Northern Finland (For researchers - Biopankki Borealis (oys.fi)) and can be used for scientific research.
